# High tPA-expression in primary melanoma of the limb correlates with good prognosis

**DOI:** 10.1054/bjoc.2000.1460

**Published:** 2000-10-26

**Authors:** C M Ferrier, S Suciu, W L van Geloof, H Straatman, A M M Eggermont, H Schraffordt Koops, B B R Kroon, F J Lejeune, U R Kleeberg, G N P van Muijen, D J Ruiter, Malignant Melanoma Cooperative Group as a side study of trial 18832

**Affiliations:** Department of PathologyDepartment of Epidemiology, University Medical Center St. Radboud, PO Box 9101, Nijmegen, HB, 6500, The Netherlands; EORTC Data Center, Av. E. Mounier 83/11, Brussels, 1200, Belgium; Department of Surgical Oncology, University Hospital Rotterdam/Daniel den Hoed Cancer Center, PO Box 5201, Rotterdam, AE, 3008, The Netherlands; Department of Surgical Oncology, University Hospital Groningen, PO Box 30001, Groningen, RB, 9700, The Netherlands; Department of Surgery, The Netherlands Cancer Institute/Antoni van Leeuwenhoek Ziekenhuis, Plesmanlaan 121, Amsterdam, CX, 1066, The Netherlands; Centre Pluridisciplinaire d'Oncologie, Centre Hospitalier Universitaire Vaudois, Lausanne, CH-1011, Switzerland; Haematologisch-onkologische Praxis Altona, Max Brauerallee 52, Hamburg, 22765, Germany

**Keywords:** plasminogen activation system, serine proteases, tPA, melanoma, prognosis, immunohistochemistry, pathology

## Abstract

To investigate whether the course of primary melanoma disease correlates with expression of the various components of the proteolytic plasminogen activation (PA) system, immunohistochemical stainings for activators of plasminogen (tissue type (tPA) and urokinase type (uPA)), inhibitors of plasminogen activation (type 1 (PAI-1) and type 2 (PAI-2)) and the receptor for uPA (uPAR) were performed on 214 routinely processed melanoma lesions. All lesions were primary cutaneous melanomas, minimally 1.5 mm thick, and derived from patients with only local disease at the moment of diagnosis (clinically stage II (T _3–4_ N _0_ M _0_), American Joint Committee on Cancer). Median patient follow-up was 6.1 years. Single variables as immunohistochemical staining results (extent of tumour cell staining, pattern of tumour cell staining and for some components also staining of stromal cells), histopathological and clinical parameters as well as treatment variables were analysed in order to assess their prognostic importance, in terms of time to recurrence, time to distant metastasis and duration of survival. The extent of tPA tumour cell positivity, categorized as 0–5%, 6–50% and 51–100%, appeared to be of importance for these end-points. Lesions with 51–100% tPA-positive tumour cells were found to have the best prognosis, whereas lesions with 6–50% tPA-positive tumour cells had the worst. Moreover, the prognostic significance of Breslow thickness, microscopic ulceration and sex was confirmed in this study. Multivariate analyses, incorporating these relevant factors, showed that the extent of tPA tumour cell positivity was an independent prognostic factor for distant metastasis-free interval (*P*= 0.012) and for the duration of survival (*P*= 0.043). © 2000 CancerResearch Campaign


British Journal of Cancer (2000) **83**(10), 1351–1359
© 2000 Cancer Research Campaign
doi: 10.1054/ bjoc.2000.1460, available online at http://www.idealibrary.com on IDEAL®

### High tPA-expression in primary melanoma of the limb correlates with good prognosis

**CM Ferrier[1], S Suciu[2], WL van Geloof[1], H Straatman[3], AMM Eggermont[4], H Schraffordt Koops[5], BBR Kroon[6], FJ Lejeune[7], UR Kleeberg[8], GNP van Muijen[1] and DJ Ruiter[1] on behalf of the European Organization for Research and Treatment of Cancer, Malignant Melanoma Cooperative Group, as a side study of trial 18832**

[1]Department of Pathology and [3]Department of Epidemiology, University Medical Center St. Radboud, PO Box 9101, 6500 HB Nijmegen, The Netherlands; [2]EORTC Data Center, Av. E. Mounier 83/11, 1200 Brussels, Belgium; [4]Department of Surgical Oncology, University Hospital Rotterdam/Daniel den Hoed Cancer Center, PO Box 5201, 3008 AE Rotterdam, The Netherlands; [5]Department of Surgical Oncology, University Hospital Groningen, PO Box 30001, 9700 RB Groningen, The Netherlands; [6]Department of Surgery, The Netherlands Cancer Institute/Antoni van Leeuwenhoek Ziekenhuis, Plesmanlaan 121, 1066 CX Amsterdam, The Netherlands; [7]Centre Pluridisciplinaire d'Oncologie, Centre Hospitalier Universitaire Vaudois, CH-1011 Lausanne, Switzerland; [8]Haematologisch-onkologische Praxis Altona, Max Brauerallee 52, 22765 Hamburg, Germany

**Summary** To investigate whether the course of primary melanoma disease correlates with expression of the various components of the proteolytic plasminogen activation (PA) system, immunohistochemical stainings for activators of plasminogen (tissue type (tPA) and urokinase type (uPA)), inhibitors of plasminogen activation (type 1 (PAI-1) and type 2 (PAI-2)) and the receptor for uPA (uPAR) were performed on 214 routinely processed melanoma lesions. All lesions were primary cutaneous melanomas, minimally 1.5 mm thick, and derived from patients with only local disease at the moment of diagnosis (clinically stage II ($T_{3–4}$ $N_0M_0$), American Joint Committee on Cancer). Median patient follow-up was 6.1 years. Single variables as immunohistochemical staining results (extent of tumour cell staining, pattern of tumour cell staining and for some components also staining of stromal cells), histopathological and clinical parameters as well as treatment variables were analysed in order to assess their prognostic importance, in terms of time to recurrence, time to distant metastasis and duration of survival. The extent of tPA tumour cell positivity, categorized as 0–5%, 6–50% and 51–100%, appeared to be of importance for these end-points. Lesions with 51–100% tPA-positive tumour cells were found to have the best prognosis, whereas lesions with 6–50% tPA-positive tumour cells had the worst. Moreover, the prognostic significance of Breslow thickness, microscopic ulceration and sex was confirmed in this study. Multivariate analyses, incorporating these relevant factors, showed that the extent of tPA tumour cell positivity was an independent prognostic factor for distant metastasis-free interval ($P$ = 0.012) and for the duration of survival ($P$ = 0.043). © 2000 Cancer Research Campaign

**Keywords**: plasminogen activation system; serine proteases; tPA; melanoma; prognosis; immunohistochemistry; pathology

It is generally accepted that proteases, primarily by degradation of the cancer-surrounding extracellular matrix, are involved in tumour cell invasion and metastasis (Reuning et al, 1998). uPA, tPA, PAI-1, PAI-2 and uPAR are members of the PA proteolytic system. The PA system controls the conversion of latent plasminogen into active plasmin. uPA and tPA activate plasminogen; PAI-1 and PAI-2 directly inhibit uPA and tPA, thereby indirectly inhibiting the formation of active plasmin. uPAR is cell membrane-bound, and by binding its ligand uPA, active plasmin can be formed in the direct environment of the cell, which contributes to directional proteolysis.

Clinical studies have shown that levels of PA components in tumour tissue of various histological types are related to prognosis. In this regard, breast carcinoma is the most extensively studied malignancy. By using the enzyme-linked immunosorbent assay (ELISA) technique, high expression levels of uPA, PAI-1 and uPAR in tumour extracts have repeatedly been reported to be related to shorter disease-free interval and overall survival, whereas high tPA levels were related to a favourable course of disease. The same trends were found for carcinoma of the gastrodigestive tract and for various other tumour types (Duffy et al, 1988; 1999; Ganesh et al, 1996; Andreasen et al, 1997; Reuning et al, 1998; de Witte et al, 1999).

In addition to ELISA techniques, immunohistochemistry (IHC) enables assessment of the presence of PA components (Ferrier et al, 1999). Advantages of IHC are that it can be performed on routinely processed paraffin-embedded tissues (Ferrier et al, 1998*a*; Ruiter et al, 1998a), and that it gives insight into the distribution of the proteins over different cell types and different areas of the tumour. By IHC, the adverse prognostic value of high uPA, PAI-1 and uPAR expression and the favourable prognostic value of high PAI-2 expression has been shown for breast cancer (Kim et al, 1997; Umeda et al, 1997), cervical cancer of the uterus (Kobayashi et al, 1994), epithelial ovarian cancer (Chambers et al, 1997; 1998), pancreatic cancer (Takeuchi et al, 1993), oesophagus cancer (Torzewski et al, 1997), gastric cancer (Ito et al, 1996; Allgayer et al, 1998), colorectal cancer (Mulcahy et al, 1994; Sato et al, 1995; Berney et al, 1998; Kim et al, 1998*b*), upper urinary

*Received 6 March 2000*
*Revised 29 June 2000*
*Accepted 11 July 2000*

*Correspondence to:* CM Ferrier

**Table 1** Patient characteristics and outcome of disease

| | Overall group | | Subgroups of tPA tumour cell positivity[a] | | | | | |
|---|---|---|---|---|---|---|---|---|
| | | | 0–5% | | 6–50% | | 51–100% | |
| | *n* = 214 | (%) | *n* = 166 | (%) | *n* = 26 | (%) | *n* = 21 | (%) |
| Age at registration (yrs) | | | | | | | | |
| Median | 50 | | 50 | | 54 | | 45 | |
| Range | 17–73 | | 17–73 | | 36–71 | | 25–70 | |
| ≤45 | 86 | (40.2) | 68 | (41.0) | 6 | (23.1) | 11 | (52.4) |
| 46–60 | 78 | (36.4) | 59 | (35.5) | 13 | (50.0) | 6 | (28.6) |
| ≥61 | 50 | (23.4) | 39 | (23.5) | 7 | (26.9) | 4 | (19.0) |
| Sex | | | | | | | | |
| Female | 147 | (68.7) | 115 | (69.3) | 17 | (65.4) | 14 | (66.7) |
| Male | 67 | (31.3) | 51 | (30.7) | 9 | (34.6) | 7 | (33.3) |
| Site | | | | | | | | |
| Upper limb | 58 | (27.1) | 44 | (26.5) | 8 | (30.8) | 6 | (28.6) |
| Lower limb | 156 | (72.9) | 122 | (73.5) | 18 | (69.2) | 15 | (71.4) |
| Treatment | | | | | | | | |
| Wide excision (WE) | 110 | (51.4) | 85 | (51.2) | 11 | (42.3) | 13 | (61.9) |
| WE plus limb perfusion | 104 | (48.6) | 81 | (48.8) | 15 | (57.7) | 8 | (38.1) |
| Elective lymph-node dissection | | | | | | | | |
| No | 156 | (72.9) | 122 | (73.5) | 19 | (73.1) | 14 | (66.7) |
| Yes | 57 | (26.6) | 43 | (25.9) | 7 | (26.9) | 7 | (33.3) |
| Unclassified | 1 | (0.5) | 1 | (0.6) | 0 | | 0 | |
| Breslow thickness | | | | | | | | |
| ≤2 mm | 44 | (20.5) | 35 | (21.1) | 4 | (15.4) | 4 | (19.0) |
| >2 mm and ≤4 mm | 114 | (53.3) | 92 | (55.4) | 13 | (50.0) | 9 | (42.9) |
| >4 mm | 56 | (26.2) | 39 | (23.5) | 9 | (34.6) | 8 | (38.1) |
| Clark's invasion level | | | | | | | | |
| 3 | 58 | (27.1) | 46 | (27.7) | 5 | (19.2) | 6 | (28.6) |
| 4 | 138 | (64.5) | 105 | (63.3) | 19 | (73.1) | 14 | (66.7) |
| 5 | 18 | (8.4) | 15 | (9.0) | 2 | (7.7) | 1 | (4.7) |
| Histological subtype | | | | | | | | |
| Superficial spreading | 93 | (43.5) | 78 | (47.0) | 8 | (30.8) | 7 | (33.3) |
| Nodular | 86 | (40.2) | 59 | (35.5) | 14 | (53.9) | 12 | (57.2) |
| Acro-lentiginous | 27 | (12.6) | 22 | (13.3) | 3 | (11.5) | 2 | (9.5) |
| Unclassified | 8 | (3.7) | 7 | (4.2) | 1 | (3.8) | 0 | |
| Microscopic ulceration | | | | | | | | |
| Absent | 128 | (59.8) | 99 | (59.6) | 13 | (50.0) | 16 | (76.2) |
| Present | 85 | (39.7) | 66 | (38.8) | 13 | (50.0) | 5 | (23.8) |
| Unclassified | 1 | (0.5) | 1 | (0.6) | 0 | | 0 | |
| Clinical course | | | | | | | | |
| Recurrence (local, regional or systemic) | | | | | | | | |
| No | 131 | (61.2) | 104 | (62.7) | 11 | (42.3) | 16 | (76.2) |
| Yes | 83 | (38.8) | 62 | (37.3) | 15 | (57.7) | 5 | (23.8) |
| Distant metastasis | | | | | | | | |
| No | 162 | (75.7) | 127 | (76.5) | 14 | (53.8) | 20 | (95.2) |
| Yes | 52 | (24.3) | 39 | (23.5) | 12 | (46.2) | 1 | (4.8) |
| Survival status | | | | | | | | |
| Alive | 161 | (75.2) | 127 | (76.5) | 14 | (53.8) | 19 | (90.5) |
| Dead[b] | 53 | (24.8) | 39 | (23.5) | 12 | (46.2) | 2 | (9.5) |

[a]One tPA score was missing for a young female with a nodular melanoma on the leg, Breslow thickness ≤2 mm, Clark level 3, ulceration present, treated by wide excision, no elective lymph-node dissection. Follow-up revealed a recurrence, no distant metastasis or death. [b]Death cases were melanoma-related with the presence of distant metastasis in 49 cases, melanoma-independent with the presence of a distant metastasis in one case, melanoma-independent without the presence of distant metastasis in two cases and without known cause in one case tract carcinoma (Nakanishi et al, 1998) and glioma (Hsu et al, 1995). However, in non-small cell lung cancer, neither ELISA nor IHC determinations for PAI-1 and uPAR were related to survival (Pappot et al, 1997).

Observations in experimental systems and on clinical material also pointed to a role for proteases in melanocytic tumour progression (see reviews by de Vries et al, 1996; Mueller, 1996; Ferrier et al, 1998*b*). The presence of PA components in common naevi, dysplastic naevi and malignant melanoma has earlier been studied by us (de Vries et al, 1994) and others (Delbaldo et al, 1994) using IHC, in situ hybridization and in situ zymography. Both found an increase of expression with higher grade of disease, and concluded that components of the PA system are markers for progression in melanocytic lesions. Also in uveal melanoma, high uPA expression was shown to be associated with progression of disease (de Vries et al, 1995).

In the present study we investigated the potential of uPA, tPA, PAI-1, PAI-2 and uPAR expression to be markers for prognosis in primary melanoma. Therefore, IHC staining was performed on 214 primary cutaneous melanomas from clinical stage II ($T_{3-4}N_0M_0$, American Joint Committee on Cancer) patients with well documented follow-up. Expression levels were correlated to:

 *© 2000 Cancer Research Campaign*

disease-free interval; time to distant metastases; and overall survival. The additive value in relation to established prognostic factors was evaluated by multivariate analysis.

### PATIENTS, MATERIALS AND METHODS

### Patients

Two hundred and fourteen clinically stage II ($T_{3–4}N_0M_0$, American Joint Committee on Cancer) patients with primary melanoma of the limb were evaluated. These patients were enrolled in an international phase III trial (no. 18832, by the European Organization for Research and Treatment of Cancer), testing the benefits of wide excision of the primary lesion plus prophylactic isolated limb perfusion with melphalan under mild hyperthermia as compared to wide excision only (Schraffordt Koops et al, 1998). Relevant inclusion criteria were tumour localization at or distal to the middle of the thigh or upper arm and a tumour-thickness of minimally 1.5 mm ($T_{3–4}$) as measured by the local pathologist. In spite of the tumour thickness requirement, three lesions had a Breslow thickness below 1.5 mm, due to discrepancies between Breslow indices as measured by the first pathologist and the reviewing pathologist. Patients who had (excision) biopsy of the primary melanoma more than 6 weeks before were not eligible for participation in the trial. For the present study, Dutch participants, included between 1986 and 1993, were studied. Complete follow-up was available for all patients. For detailed patient characteristics, see Table 1.

### Tissues

Paraffin blocks were collected from 45 different departments of pathology. All tissues had been routinely processed. Of each primary melanoma, the tissue block containing the thickest portion of the lesion was selected for investigation.

### Immunohistochemistry

Of each lesion 4 μm thick sections were stained with polyclonal antibodies against uPA (rabbit antibody, DAKO, Glostrup, Denmark), tPA (goat antibody no. 387, American Diagnostica, Greenwich CT, USA), PAI-1 (rabbit antibody, Department of Experimental and Chemical Endocrinology, University Medical Center St Radboud, Nijmegen, The Netherlands (Grebenschikov et al, 1997)), PAI-2 (goat antibody, Behring Werke AG, Marburg, Germany) and uPAR (rabbit antibody, Finsen Laboratory, Copenhagen, Denmark (Rønne et al, 1995)). To check sensitivity and specificity, stainings with other antibodies against tPA (rabbit polyclonal antibody, Department of Experimental and Chemical Endocrinology, University Medical Center St Radboud, Nijmegen, The Netherlands (Grebenschikov et al, 1997)), PAI-2 (goat polyclonal antibody, Institute for Immunology, University Hospital Heidelberg, Germany (Schaefer et al, 1996)) and uPAR (monoclonal antibody R2, Finsen Laboratory (Rønne et al, 1991)), and with two other antibodies against PAI-1 (monoclonal antibody Clone 1, Monozyme, Hoersholm, Denmark; monoclonal antibody no. 380, American Diagnostica) were compared on more than 100 lesions.

IHC procedures were performed as described previously (Ferrier et al, 1998*a*). In brief, firstly the epitopes for antibody recognition were retrieved by microwave-heating (for tPA, PAI-1 and PAI-2) or by protease digestion (for uPA and uPAR). Then, the sections were stained by a three-step procedure using biotinylated secondary antibodies in combination with alkaline phosphatase-labelled avidin–biotin complex (Vector Laboratories, Burlingame CA, USA). Secondary antibodies were goat anti-rabbit immunoglobulin (Vector Laboratories), donkey anti-mouse immunoglobulin (Jackson, West Grove PA, USA), and donkey anti-goat immunoglobulin (Jackson). Vector Red (Vector Laboratories) was used as substrate to enable a clear distinction between melanin granules and staining signal.

For each component, positive and negative tissue controls (Ferrier et al, 1998*a*), plus cancer lesions known to contain both antigen-positive and antigen-negative areas were included in each staining session. Negative staining controls were performed on a subset of antigen-positive primary melanoma lesions by replacing monoclonal antibodies with an isotype-matched irrelevant antibody (DAKGO1, Dako) and by replacing polyclonal antibodies with a primary antibody dilution which was affinity-absorbed with purified antigen and by ommission of the first antibody.

### Scoring

Sections stained with polyclonal antibodies that had been used on the total group of 214 lesions were scored. First, to advance standardization, scores were assessed by joint judgement (CMF, GNPvM and DJR). After these sessions, sections were primarily scored by one observer (CMF), with joint scoring sessions for 20–30% of the sections, including random cases and all cases difficult to score. Melanoma cells were scored according to the percentage of positive cells in six categories: 0–1%, 2–5%, 6–25%, 26–50%, 51–75% or 76–100%. The distribution of melanoma cell staining was judged as: diffuse positivity, involving large continuous parts of the tumour; scattered positivity of single cells; or focal positivity of clusters of cells. In case of focal positivity, the involvement of junctional/subepidermal, central and/or frontal nests was noted. For stromal staining, the type of positive cells (fibroblast-like cells, macrophages, endothelial cells) was recorded and an estimate of the quantity of positive cells was made (0 = nil; 1 = a few cells; 2 = several clusters of cells; 3 = abundant stromal-cell positivity). Only stainings exceeding weak intensity were considered as positive.

### Statistical methods

For statistical evaluation, percentage-classes reflecting the extent of tumour cell positivity were taken together to form the categories 0–5%, 6–50% and 51–100% positive tumour cells.

Associations between the extent of tumour cell positivity of different components with each other were assessed by the Spearman's rank correlation coefficient. Relation between on the one side percentage categories (0–5%, 6–50% and 51–100%) and staining patterns of the investigated components, and on the other side established prognostic variables such as Breslow thickness, ulceration and sex, were determined by Spearman's rank correlation and/or Kruskall–Wallis test.

Starting point for the different analyses was the date of registration in clinical trial no. 18832. The end-point for relapse-free interval was the date of first progression, whether local relapse, in-transit metastasis, regional lymph-node metastasis or distant metastasis. The end-point for distant metastasis-free interval was the diagnosis of distant metastasis. The end-point for the duration

© 2000 Cancer Research Campaign

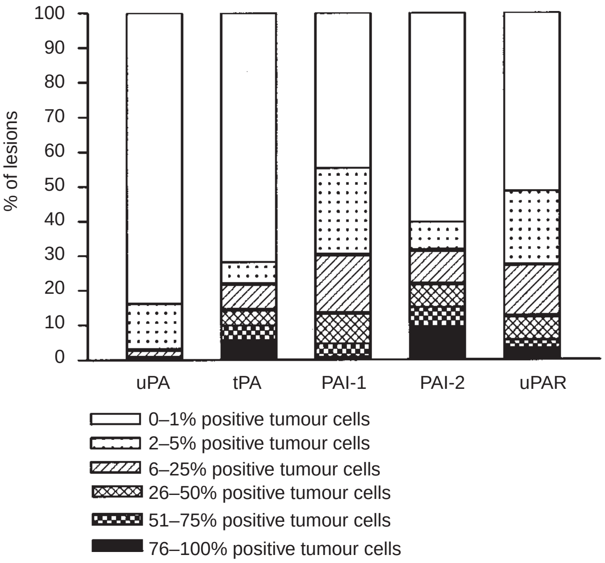

**Figure 1** Percentage of immunohistochemically stained tumour cells per lesion expressed as a percentage of the total number of stained lesions

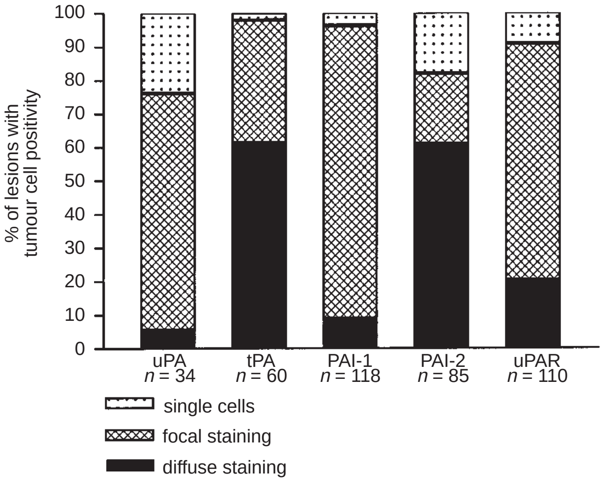

**Figure 2** Staining patterns expressed as a percentage of the positive lesions (lesions with tumour cell positivity of 1–100%).

of survival was the date of death, whatever cause. Patients still alive were considered as censored observations at the date of last follow-up. The prognostic impact of a given variable, like sex, age, site of primary melanoma, Breslow thickness, ulceration, Clark's level of invasion, type of melanoma, treatment randomized, elective lymph-node dissection, variables resulting from the IHC stainings, was first analysed univariately by estimation of the relative risks of having an event (recurrence of disease, distant metastasis, death) per unit time, along with its 95% confidence interval by applying the Cox proportional hazards method (Cox, 1972). *P* values were calculated by the –2log likelihood test. For tPA, in addition, survival curves were computed using the Kaplan–Meier technique (Kaplan and Meier, 1958). Differences between curves were tested using the log-rank test. Subsequently, independence of prognostic factors was assessed. Therefore, each variable univariately reaching significance ($P \leq 0.05$) for one of the end-points was studied in a multivariate Cox proportional hazards model.

### RESULTS

### Staining results

Staining results obtained by different antibodies against tPA, PAI-1, PAI-2 and uPAR yielded concordant patterns per component with slight variations in intensities. Frequency distributions of the extent of tumour cell staining and of the staining patterns are summarized in Figures 1 and 2. PAI-1 showed a particular staining pattern; focal staining with participation of junctional/subepidermal tumour nests was seen in the majority of positive cases. 103 of 118 PAI-1-positive lesions had focal tumour cell staining; the junctional/subepidermal region was involved in 100 cases, central tumour parts in 33 cases and the frontal region in 17 cases. For tPA, because of its relation to outcome of disease (see univariate and multivariate analyses), frequencies of the extent of tumour cell positivity are depicted for patient and treatment variables (Table 1). Representative IHC staining results are shown in Figure 3.

Spearman's rank correlation coefficients between the extent of tumour cell expression of the different components were weak, although significant between uPAR on the one side and uPA, tPA and PAI-2 on the other side ($r = 0.21$, 0.17 and 0.16, respectively), and between PAI-2 and tPA ($r = 0.34$).

Stromal cell staining of macrophages, fibroblast-like cells and endothelial cells was usually very limited; only for PAI-2 and uPAR did more than 10 lesions have stromal cell positivity which exceeded a few cells.

### Correlation of new variables with established prognostic clinicopathological variables

The percentage of PAI-1-positive tumour cells was negatively correlated with Breslow thickness (Spearman's rank correlation coefficient = –0.18, $P = 0.009$). Other significant correlations with Breslow thickness, sex or ulceration were not found, neither for the percentage of positive cells nor for staining pattern.

### Prognostic factor analyses

#### *Established prognostic factors*

As shown in Table 2, Breslow thickness and microscopic ulceration were important prognostic factors for each of the end-points time to recurrence of disease, time to distant metastasis and overall survival. Sex, site and randomized treatment appeared to be significant for one of these endpoints; subtype, Clark's level, age and elective lymph-node dissection did not reach the significance level in any analysis.

#### *uPA, PAI-1, PAI-2, uPAR*

Regarding disease-free interval, time to distant metastasis and survival, Cox proportional hazards analysis did not yield significant risk ratios for either percentage of positive tumour cells or the pattern of tumour cell positivity. For PAI-2 and uPAR, the extent of stromal cell positivity was statistically evaluated, since for these components more than ten lesions had substantial stromal cell positivity. However, no groups with differences in prognosis were found.

 © 2000 Cancer Research Campaign

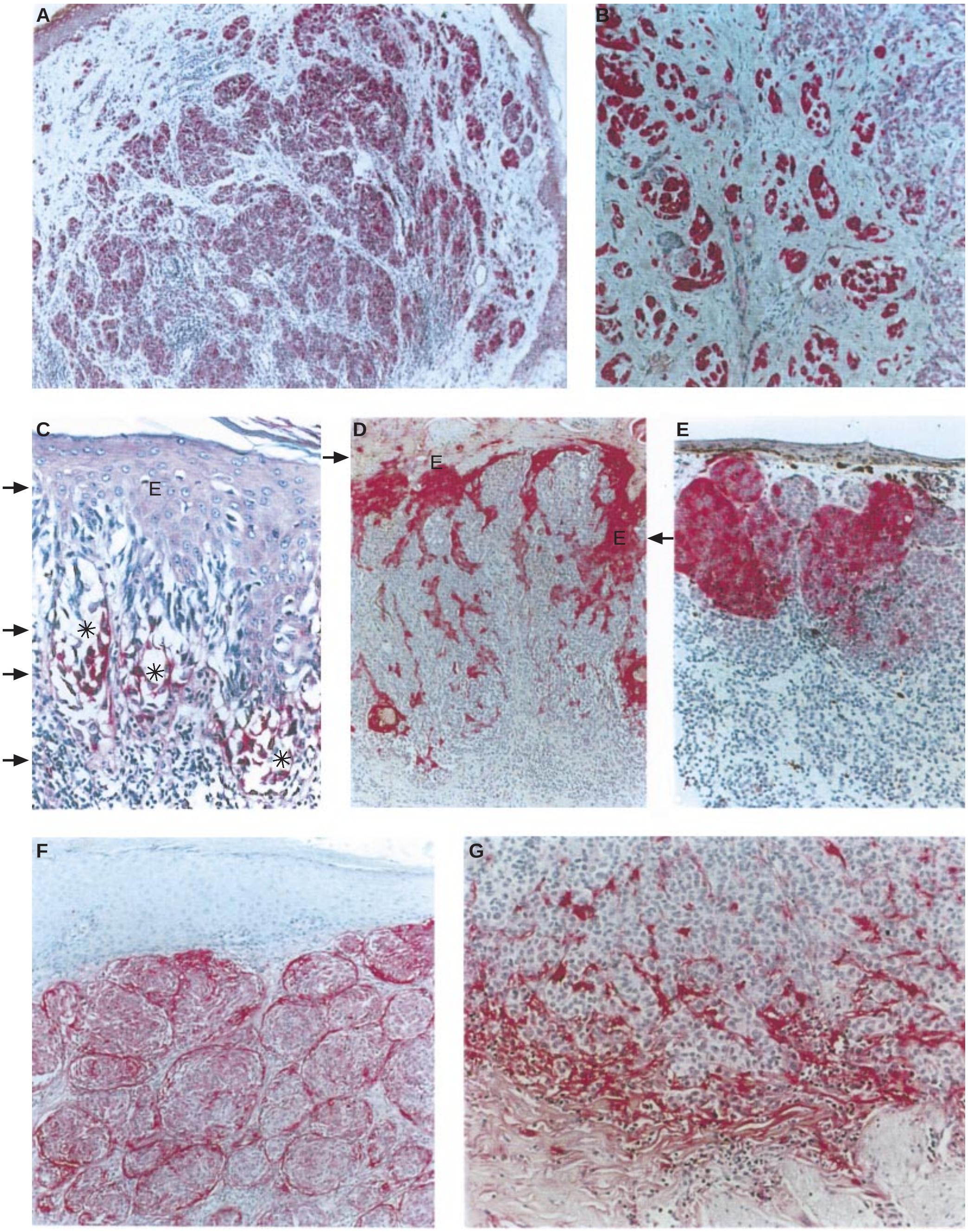

**Figure 3** Primary melanoma lesions stained for tPA (**A**, **B**), uPA (**C**), PAI-2 (**D**), PAI-1 (**E**), and uPAR (**F**, **G**). (**A**) Lesion with complete tPA tumour cell positivity. (**B**) Strong tPA-positive tumour nests and adjacent weakly-positive tumour nests. (**C**) Strong uPA-positivity of tumour nests from the horizontal growth phase (asterisks), and weak positivity of the epidermis (*E*). (**D**) Strong PAI-2-positivity of the epidermis and epidermal extensions (*E*) and negativity of melanoma cells. (**E**) PAI-1 positivity of subepidermal melanoma nests, a staining pattern frequently encountered for PAI-1. (**F**) A lesion with diffuse uPAR-positivity of tumour nests and of stromal cells surrounding these nests. (**G**) Lesion with uPAR-staining of frontally located stromal cells. Only a very limited number of tumour cells are positive

### *tPA*

Figure 4 shows the prognostic impact of tPA on the three end-points relapse-free interval, distant metastasis-free interval and duration of survival. In each of these analyses, patients with lesions having tumour cell positivity in the intermediate category (6–50%) had the worst prognosis, whereas those with the highest percentage of positivity (51–100%) had the best outcome. Table 2 shows the estimated relative risks along with the confidence intervals corresponding to these two groups of patients as compared with the one with the lowest degree of positivity (0–5%). The Cox proportional hazards model showed that if other factors, like Breslow thickness, ulceration, sex, site and allocated treatment, are taken into consideration, the degree of tPA-positivity remained of prognostic importance for the time to distant metastasis ($P$ = 0.012) and for the duration of survival ($P$ = 0.043). In these two analyses, the relative risk estimates of the middle group (6–50% positivity) compared with the 0–5% group equalled 1.9 and the relative risk estimates of the highest group (51–100% positivity)

*© 2000 Cancer Research Campaign* 

**Table 2** Results of univariate and multivariate analyses of established prognostic variables, treatment variables and tPA tumour cell positivity

| | Relapse-free interval | | | | Distant metastasis-free interval | | | | Overall survival | | | |
|---|---|---|---|---|---|---|---|---|---|---|---|---|
| | Univariate | | Multivariate | | Univariate | | Multivariate | | Univariate | | Multivariate | |
| | RR | $P^a$ | RR | $P^b$ | RR | $P^a$ | RR | $P^b$ | RR | $P^a$ | RR | $P^b$ |
| Breslow (mm) | | < 0.001 | | | | 0.011 | | | | <0.001 | | |
| 2–4 vs ≤ 2 | 2.6 | | 2.3 | | 3.0 | | 2.6 | | 3.9 | | 3.4 | |
| > 4 vs ≤ 2 | 4.0 | | 3.0 | | 5.7 | | 4.4 | | 7.9 | | 6.2 | |
| Ulceration | | < 0.001 | | | | 0.022 | | | | 0.036 | | |
| Pres vs abs | 2.4 | | 1.9 | | 1.9 | | 1.3 | | 1.8 | | 1.2 | |
| Sex | | 0.059 | | | | 0.085 | | | | 0.031 | | |
| Male vs fem | 1.5 | | 1.7 | | 1.6 | | 1.6 | | 1.8 | | 1.8 | |
| Age | | 0.965 | | | | 0.742 | | | | 0.492 | | |
| 46–60 vs ≤ 45 | 0.9 | | | | 1.2 | | | | 1.3 | | | |
| ≥ 61 vs ≤ 45 | 1.0 | | | | 1.1 | | | | 1.2 | | | |
| Site | | 0.009 | | | | 0.179 | | | | 0.087 | | |
| Leg vs arm | 2.0 | | 2.0 | | 1.6 | | 1.4 | | 1.8 | | 1.6 | |
| Treatment | | 0.528 | | | | 0.039 | | | | 0.102 | | |
| ILP vs contr | 1.1 | | 1.1 | | 1.8 | | 1.6 | | 1.6 | | 1.4 | |
| ELND | | 0.058 | | | | 0.916 | | | | 0.801 | | |
| Yes vs no | 0.6 | | | | 1.0 | | | | 0.9 | | | |
| Subtype | | 0.229 | | | | 0.749 | | | | 0.784 | | |
| NM vs SSM | 1.5 | | | | 1.2 | | | | 1.2 | | | |
| ALM vs SSM | 1.1 | | | | 0.9 | | | | 0.9 | | | |
| Clark's level | | 0.428 | | | | 0.505 | | | | 0.288 | | |
| 4 vs 3 | 1.0 | | | | 0.9 | | | | 0.9 | | | |
| 5 vs 3 | 1.7 | | | | 1.6 | | | | 1.9 | | | |
| tPA (%-categ) | | 0.041 | | 0.157 | | 0.003 | | 0.012 | | 0.013 | | 0.043 |
| 6–50 vs 0–5 | 1.8 (1.04–3.23) | | 1.5 (0.82–2.63) | | 2.3 (1.23–4.48) | | 1.9 (1.00–3.76) | | 2.3 (1.23–4.48) | | 1.9 (1.00–3.76) | |
| > 51 vs 0–5 | 0.6 (0.23–1.41) | | 0.6 (0.23–1.43) | | 0.2 (0.03–1.41) | | 0.2 (0.03–1.43) | | 0.4 (0.10–1.64) | | 0.4 (0.09–1.64) | |

RR = relative risk value (and its 95% confidence interval); [a]*P*-values of univariate proportional hazards method, computed using the –2log likelihood test; [b]*P*-values given by the –2log likelihood test, assessing the loss of discard of the tPA variable from the model in the multivariate proportional hazards method; ILP = isolated limb perfusion; ELND = elective lymph-node dissection; SSM = superficial spreading melanoma; NM = nodular melanoma; ALM = acro-lentiginous melanoma

*© 2000 Cancer Research Campaign*

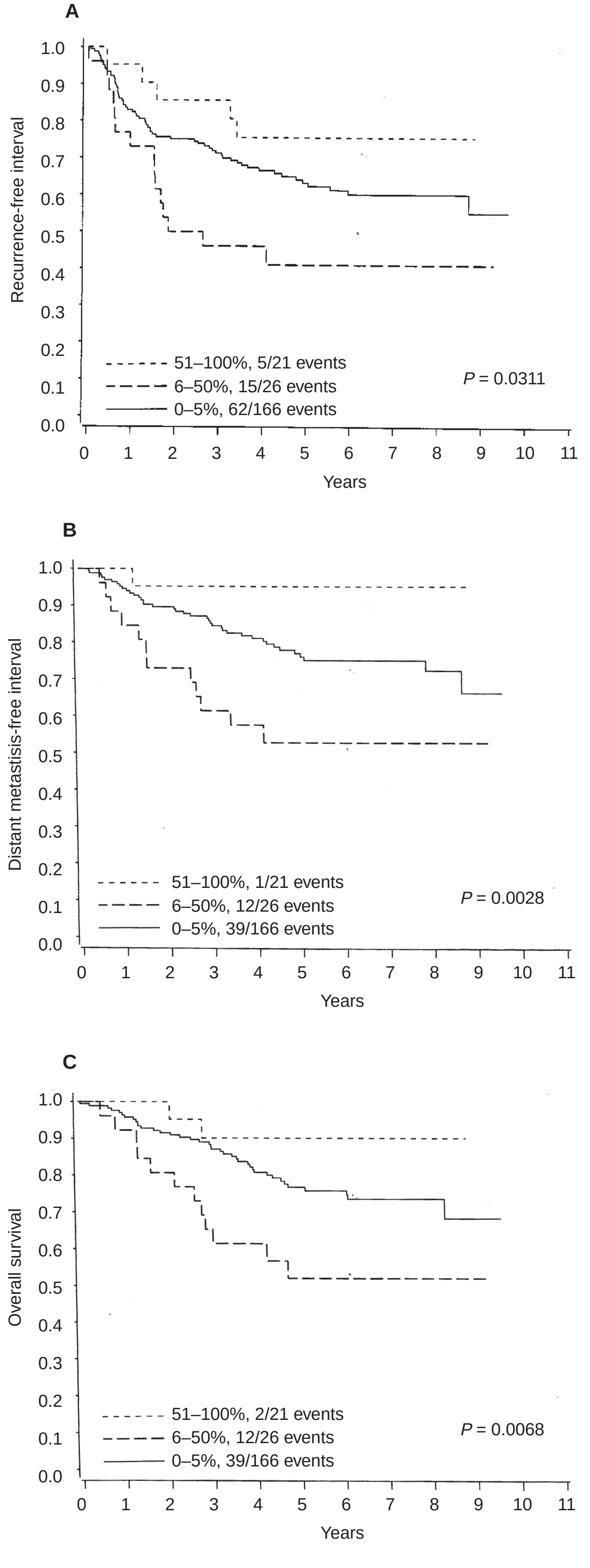

**Figure 4** Kaplan–Meier curves for recurrence-free interval. (**A**), distant metastasis-free interval (**B**) and overall survival (**C**). *P*-values were calculated by the log-rank test.

were 0.2 and 0.4 respectively. When another approach was chosen, including in the multivariate model only those variables that were significant in univariate analysis for that specific end-point, the relative risks for tPA-positivity changed slightly. Furthermore, the corresponding *P*-values decreased, which emphasized even more the prognostic importance of tPA-positivity (results not shown).

### DISCUSSION

Former studies on frozen sections of melanomas found components of the PA system to be associated with melanoma progression (Delbaldo et al, 1994; de Vries et al, 1994). This stimulated us to perform the present study based on primary melanomas of the limb from patients who had been prospectively followed in the framework of an EORTC trial (no. 18832). To that end, 214 routinely processed paraffin-embedded primary melanoma lesions were IHC stained.

Differences in uPA, tPA, PAI-1 and PAI-2 expression patterns between former studies (Delbaldo et al, 1994; de Vries et al, 1994) and the present study may be explained by the relatively small number of advanced primary melanomas investigated in the previous studies, which makes results less suitable for generalization. Moreover, the former studies were performed on frozen tissue samples, which are mostly of limited volume, but which have better antigen preservation. Since especially uPA was previously found to be sensitive to fixation time (Ferrier et al, 1998*a*), this should be kept in mind considering the low percentage of uPA-positive lesions found in the present study. For PAI-1, we found in an earlier study that this antigen on stromal cells cannot be as reliably unmasked as on tumour cells (Ferrier et al, 1998*a*).

Remarkably, extensive tPA-expression of melanoma cells was related to favourable outcome, whereas for none of the components expression was related to unfavourable outcome of disease, stressing the point that a progression marker is not automatically a prognostic parameter for aggressiveness of disease (Ruiter and van Muijen, 1998b).

The present study showed best prognosis for the group with abundant tPA tumour cell positivity (51–100%), worse outcome for the limited tPA tumour cell positive group (0–5%), and, strikingly, worst outcome in patients with intermediate tPA tumour cell expression (6–50%). No relation between staining pattern and course of disease was found. tPA tumour cell positivity was an independant prognostic factor for distant metastasis-free interval and overall survival in a multivariate model including other prognostic parameters like Breslow thickness, ulceration, sex, site and treatment.

Attempting to exclude the possibility of a statistical artefact, analyses for tPA were repeated in another small ($n$ = 24) group of European patients that were enrolled in another EORTC trial (no. 18871). This limited study confirmed the results described in this work (results not shown).

The fact that high tPA correlates with good prognosis is not unexpected. This correlation was also found for other types of tumour (Duffy et al, 1998; Jänicke et al, 1991; Yamashita et al, 1993; 1995; Bindal et al, 1994; Ganesh et al, 1996; Duggan et al, 1997; Ruppert et al, 1997; Kim et al, 1998*a*; de Witte et al, 1999), mostly by using the ELISA technique. Regarding the role of tPA in melanoma invasion, in vitro experiments and animal experimental observations have shown equivocal results (for reviews see de Vries et al, 1996; Ferrier et al, 1998*b*).

© 2000 Cancer Research Campaign

Host protective effects of tPA may be caused by dissolution of the fibrin coating of tumour emboli, as previously discussed by Yamashita et al (1993). Tumour cell-generated thrombin causes intravascular tumour cells to form microthrombi consisting of tumour cells, platelets and fibrin. These microthrombi secure the arrest of cancer cells in the capillaries and may lead to diapedesis through the capillary wall into the tissues (Rickles and Edwards, 1983; Markus, 1984; Francis et al, 1998; Engelberg, 1999). Fibrin coating is found to protect tumour cells against the lytic effects of natural killer cells and lymphokine-activated killer cells (Gunji and Gorelik, 1988; Cardinali et al, 1990). Fibrinolysis by circulating tumour cells, for example by means of fibrin-activated tPA, with consequent dissolution of fibrin deposits around embolized peripheral tumour cells, may decrease the chances of these cells to implant and to form metastases. Another host-protective effect may be the formation of angiostatin, a tumour-derived angiogenesis inhibitor (O'Reilly et al, 1996). A recent study of our group revealed that angiostatin formation by human melanoma cell lines is basically tPA-dependent (Westphal et al, 2000).

A tumour metastasis-promoting effect of tPA may be plasmin-mediated degradation of extracellular matrix. This may facilitate local invasion and release of tumour cells into the circulation. However, several experimental reports indicate that melanoma progression appears to be uPA rather than tPA-related (Quax et al, 1991; Meissauer et al, 1992; Huijzer et al, 1995; Shapiro et al, 1996). A possible stimulatory effect of tPA on angiogenesis might also enhance metastatic potential (Sato et al, 1993; Hu et al, 1994; Welling et al, 1996), although no influence of tPA on angiogenesis was found by others (Takei et al, 1995; Lansink et al, 1998; Sakamoto et al, 1998).

Based on the foregoing, it may be speculated that a shifting balance of metastatic and anti-metastatic effects may act simultaneously with a different predominant effect depending on the level of tPA. A more or less comparable finding was reported by Yamashita et al (1993), who found that a breast cancer group that remained distant metastasis-free had highest tPA-antigen levels and tPA-activity. Most seriously diseased patients (lung or both lung and bone metastases) had intermediate levels and the group with only bone metastases (bone metastases were explained to often remain localized in the bone for a long time without any further evidence of metastases) had lowest levels.

The apparently complex association between tPA-expression and the outcome of disease in case of melanoma needs to be validated in further series. If the present results are confirmed, it may be considered to offer adjuvant therapy to patients with tPA-based unfavourable prognosis, whereas for patients with high tPA-expressions, and therefore favourable prospects, no additional therapy would be indicated.

### ACKNOWLEDGEMENTS

This study was supported by the Dutch Cancer Society (project number NKB-KWF 94–722). We gratefully acknowledge pathologists from the Netherlands who provided us with tissue blocks from patients that had participated in trial no. 18832. We thank Mrs LGM Verhoeven for coordinating the transfer of tissue blocks, Dr N Grebenschikov (Department of Chemical Endocrinology, University Medical Center St Radboud, Nijmegen, The Netherlands) for the antibodies against tPA and PAI-1, Dr J Askaa (DAKO, Glostrup, Denmark) for the antibody against uPA, Dr E Schüler (Behring Werke AG, Marburg, Germany) and Prof MD Kramer (Institute for Immunology, Laboratory for Immunopathology, University Hospital of Heidelberg, Germany) for the antibodies against PAI-2 and Dr N Brünner (Finsen Laboratory, Copenhagen, Denmark) for the antibodies against uPAR.

### REFERENCES

Allgayer H, Babic R, Grützner KU, Beyer BCM, Tarabichi A, Schildberg FW and Heiss MM (1998) Tumor-associated proteases and inhibitors in gastric cancer: analysis of prognostic impact and individual risk protease patterns. *Clin Exp Metastasis* **16**: 62–73

Andreasen PA, Kjøller L, Christensen L and Duffy MJ (1997) The urokinase-type plasminogen activator system in cancer metastasis: a review. *Int J Cancer* **72**: 1–22

Berney CR, Yang J, Fisher RJ, Russell PJ and Crowe PJ (1998) Correlates of urokinase-type plasminogen activator in colorectal cancer: positive relationship with nm23 and c-erbB-2 protein expression. *Oncol Res* **10**: 47–54

Bindal AK, Hammoud M, Shi WM, Wu SZ, Sawaya R and Rao JS (1994) Prognostic significance of proteolytic enzymes in human brain tumors. *J Neurooncol* **22**: 101–110

Cardinali M, Uchino R and Chung SI (1990) Interaction of fibrinogen with murine melanoma cells: covalent association with cell membranes and protection against recognition by lymphokine-activated killer cells. *Cancer Res* **50**: 8010–8016

Chambers SK, Ivins CM and Carcangiu ML (1997) Expression of plasminogen activator inhibitor-2 in epithelial ovarian cancer: a favorable prognostic factor related to the actions of CSF-1. *Int J Cancer* **74**: 571–575

Chambers SK, Ivins CM and Carcangiu ML (1998) Plasminogen activator inhibitor-1 is an independent poor prognostic factor for survival in advanced stage epithelial ovarian cancer patients. *Int J Cancer* **79**: 449–454

Cox DR (1972) Regression models and life-tables. *J R Stat Soc B* **34**: 187–220

Delbaldo C, Masouye I, Saurat J-H, Vassalli J-D and Sappino A-P (1994) Plasminogen activation in melanocytic neoplasia. *Cancer Res* **54**: 4547–4552

de Vries TJ, Quax PHA, Denijn M, Verrijp KN, Verheijen JH, Verspaget HW, Weidle UH, Ruiter DJ and van Muijen GNP (1994) Plasminogen activators, their inhibitors, and urokinase receptor emerge in late stages of melanocytic tumor progression. *Am J Pathol* **144**: 70–81

de Vries TJ, Mooy CM, van Balken MR, Luyten GPM, Quax PHA, Verspaget HW, Weidle UH, Ruiter DJ and van Muijen GNP (1995) Components of the plasminogen activation system in uveal melanoma – a clinico-pathological study. *J Pathol* **175**: 59–67

de Vries TJ, van Muijen GNP and Ruiter DJ (1996) The plasminogen activation system in melanoma cell lines and in melanocytic lesions. *Melanoma Res* **6**: 79–88

de Witte JH, Sweep CGJ, Klijn JGM, Grebenschikov N, Peters HA, Look MP, van Tienoven ThH, Heuvel JJTM, Bolt-de Vries J, Benraad ThJ and Foekens JA (1999) Prognostic value of tissue-type plasminogen activator (tPA) and its complex with the type-1 inhibitor (PAI-1) in breast cancer. *Br J Cancer* **80**: 286–294

Duffy MJ, O'Grady P, Devaney L, O'Siorain L, Fennelly JJ and Lijnen HR (1988) Tissue-type plasminogen activator, a new prognostic marker in breast cancer. *Cancer Res* **48**: 1348–1349

Duffy MJ, Maguire TM, McDermott EW and O'Higgins N (1999) Urokinase plasminogen activator: a prognostic marker in multiple types of cancer. *J Surg Oncol* **71**: 130–135

Duggan C, Kramer MD, Barnes C, Elvin P, McDermott E, O'Higgins N and Duffy MJ (1997) Plasminogen activator inhibitory type 2 in breast cancer. *Br J Cancer* **76**: 622–627

Engelberg H (1999) Actions of heparin that may affect the malignant process. *Cancer* **85**: 257–272

Ferrier CM, van Geloof WL, de Witte JH, Kramer MD, Ruiter DJ and van Muijen GNP (1998*a*) Epitopes of components of the plasminogen activation system are re-exposed in formalin-fixed paraffin sections by different retrieval techniques. *J Histochem Cytochem* **46**: 469–476

Ferrier CM, van Muijen GNP and Ruiter DJ (1998*b*) Proteases in cutaneous melanoma. *Ann Med* **30**: 431–442

Ferrier CM, de Witte JH, Straatman H, van Tienoven DH, van Geloof WL, Rietveld FJR, Sweep CGJ, Ruiter DJ and van Muijen GNP (1999) Comparison of immunohistochemistry with immunoassay (ELISA) for the detection of components of the plasminogen activation system in human tumour tissue. *Br J Cancer* **79**: 1534–1541

 © 2000 Cancer Research Campaign

Francis JL, Biggerstaff J and Amirkhosravi A (1998) Hemostasis and malignancy. *Semin Thromb Hemost* **24**: 93–109

Ganesh S, Sier CFM, Heerding MM, van Krieken JHJM, Griffioen G, Welvaart K, van de Velde CJH, Verheijen JH, Lamers CBHW and Verspaget HW (1996) Prognostic value of the plasminogen activation system in patients with gastric carcinoma. *Cancer* **77**: 1035–1043

Grebenschikov N, Geurts-Moespot A, de Witte H, Heuvel J, Leake R, Sweep F and Benraad T (1997) A sensitive and robust assay for urokinase and tissue-type plasminogen activators (uPA and tPA) and their inhibitor type I (PAI-1) in breast tumor cytosols. *Int J Biol Markers* **12**: 6–14

Gunji Y and Gorelik E (1988) Role of fibrin coagulation in protection of murine tumor cells from destruction by cytotoxic cells. *Cancer Res* **48**: 5216–5221

Hsu DW, Efird JT and Hedley-Whyte ET (1995) Prognostic role of urokinase-type plasminogen activator in human gliomas. *Am J Pathol* **147**: 114–123

Hu G-F, Riordan JF and Vallee BL (1994) Angiogenin promotes invasiveness of cultured endothelial cells by stimulation of cell-associated proteolytic activities. *Proc Natl Acad Sci USA* **91**: 12096–12100

Huijzer JC, Uhlenkott CE and Meadows GG (1995) Differences in expression of metalloproteinases and plasminogen activators in murine melanocytes and B16 melanoma variants: lack of association with in vitro invasion. *Int J Cancer* **63**: 92–99

Ito H, Yonemura Y, Fujita H, Tsuchihara K, Kawamura T, Nojima N, Fujimura T, Nose H, Endo Y and Sasaki T (1996) Prognostic relevance of urokinase-type plasminogen activator (uPA) and plasminogen activator inhibitors PAI-1 and PAI-2 in gastric cancer. *Virchows Arch* **427**: 487–496

Jänicke F, Schmitt M and Graeff H (1991) Clinical relevance of the urokinase-type and tissue-type plasminogen activators and of their type 1 inhibitor in breast cancer. *Semin Thromb Hemost* **17**: 303–312

Kaplan EL and Meier P (1958) Nonparametric estimation from incomplete observations. *J Am Stat Assoc* **53**: 457–481

Kim SJ, Shiba E, Taguchi T, Watanabe T, Tanji Y, Kimoto Y, Izukura M and Takai S-I (1997) Urokinase type plasminogen activator receptor is a novel prognostic factor in breast cancer. *Anticancer Res* **17**: 1373–1378

Kim SJ, Shiba E, Kobayashi T, Yayoi E, Furukawa J, Takatsuka Y, Shin E, Koyama H, Inaji H and Takai S (1998*a*) Prognostic impact of urokinase-type plasminogen activator (PA), PA-inhibitor type-1, and tissue-type PA antigen levels in node-negative breast cancer; a prospective study on multicenter basis. *Clin Cancer Res* **4**: 177–182

Kim SJ, Shiba E, Tsukamoto F, Izukura M, Taguchi T, Yoneda K, Tanji Y, Kimoto Y and Takai S-I (1998*b*) The expression of urokinase type plasminogen activator is a novel prognostic factor in Dukes B and C colorectal cancer. *Oncol Rep* **5**: 431–435

Kobayashi H, Fujishiro S and Terao T (1994) Impact of urokinase-type plasminogen activator and its inhibitor type 1 on prognosis in cervical cancer of the uterus. *Cancer Res* **54**: 6539–6548

Lansink M, Koolwijk P, van Hinsbergh V and Kooistra T (1998) Effect of steroid hormones and retinoids on the formation of capillary-like tubular structures of human microvascular endothelial cells in fibrin matrices is related to urokinase expression. *Blood* **92**: 927–938

Markus G (1984) The role of hemostasis and fibrinolysis in the metastatic spread of cancer. *Semin Thromb Hemost* **10**: 61–70

Meissauer A, Kramer MD, Schirrmacher V and Brunner G (1992) Generation of cell surface-bound plasmin by cell-associated urokinase-type or secreted tissue-type plasminogen activator: a key event in melanoma cell invasiveness in vitro. *Exp Cell Res* **199**: 179–190

Mueller BM (1996) Different roles for plasminogen activators and metalloproteinases in melanoma metastasis. *Curr Top Microbiol Immunol* **213**: 65–80

Mulcahy HE, Duffy MJ, Gibbons D, McCarthy P, Parfrey NA, O'Donoghue DP and Sheahan K (1994) Urokinase-type plasminogen activator and outcome in Dukes' B colorectal cancer. *Lancet* **344**: 583–584

Nakanishi K, Kawai T, Torikata C, Aurues T and Ikeda T (1998) Urokinase-type plasminogen activator, its inhibitor, and its receptor in patients with upper urinary tract carcinoma. *Cancer* **82**: 724–732

O'Reilly MS, Holmgren L, Cheng C and Folkman J (1996) Angiostatin induces and sustains dormancy of human primary tumors in mice. *Nat Med* **2**: 689–692

Pappot H, Guldhammer Skov B, Pyke C and Grøndahl-Hansen J (1997) Levels of plasminogen activator inhibitor type 1 and urokinase plasminogen activator receptor in non-small cell lung cancer as measured by quantitative ELISA and semiquantitative immunohistochemistry. *Lung Cancer* **17**: 197–209

Quax PHA, van Muijen GNP, Weening-Verhoeff EJD, Lund LR, Danø K, Ruiter DJ and Verheijen JH (1991) Metastatic behavior of human melanoma cell lines in nude mice correlates with urokinase-type plasminogen activator, its type-1 inhibitor, and urokinase-mediated matrix degradation. *J Cell Biol* **115**: 191–199

Reuning U, Magdolen V, Wilhelm O, Fischer K, Lutz V, Graeff H and Schmitt M (1998) Multifunctional potential of the plasminogen activation system in tumor invasion and metastasis (review). *Int J Oncol* **13**: 893–906

Rickles FR and Edwards RL (1983) Activation of blood coagulation in cancer: Trousseau's syndrome revisited. *Blood* **62**: 14–31

Rønne E, Behrendt N, Ellis V, Ploug M, Danø K and Høyer-Hansen G (1991) Cell-induced potentiation of the plasminogen activation system is abolished by a monoclonal antibody that recognizes the $NH_2$-terminal domain of the urokinase receptor. *FEBS Lett* **288**: 233–236

Rønne E, Høyer-Hansen G, Brünner N, Pederson H, Rank F, Osborne CK, Clark GM, Danø K and Grøndahl-Hansen J (1995) Urokinase receptor in breast cancer tissue extracts. Enzyme-linked immunosorbent assay with a combination of mono- and polyclonal antibodies. *Breast Cancer Res Treat* **33**: 199–207

Ruiter DJ, Ferrier CM, van Muijen GNP, Henzen-Logmans SC, Kennedy S, Kramer MD, Nielsen BS and Schmitt M (1998a) Quality control of immunohistochemical evaluation of tumour-associated plasminogen activators and related components. *Eur J Cancer* **34**: 1334–1340

Ruiter DJ and van Muijen GNP (1998b) Markers of melanocytic tumour progression. *J Pathol* **186**: 340–342

Ruppert C, Ehrenforth S, Scharrer I and Halberstadt E (1997) Protease levels in breast, ovary, and other gynecological tumor tissues: prognostic importance in breast cancer. *Cancer Detect Prev* **21**: 452–459

Sakamoto T, Spee C, Scuric Z, Gordon EM, Hinton DR, Anderson WF and Ryan SJ (1998) Ability of retroviral transduction to modify the angiogenic characteristics of RPE cells. *Graefes Arch Clin Exp Ophthalmol* **236**: 220–229

Sato Y, Okamura K, Morimoto A, Hamanaka R, Hamaguchi K, Shimada T, Ono M, Kohno K, Sakata T and Kuwano M (1993) Indispensable role of tissue-type plasminogen activator in growth factor-dependent tube formation of human microvascular endothelial cells in vitro. *Exp Cell Res* **204**: 223–229

Sato T, Nishimura G, Yonemura Y, Nojima N, Ninomiya I, Fujimura T, Sugiyama K, Miwa K, Miyazaki I, Nonomura A and Yamaguchi Y (1995) Association of immuno-histochemical detection of urokinase-type plasminogen activator with metastasis and prognosis in colorectal cancer. *Oncology* **52**: 347–352

Schaefer BM, Maier K, Eickhoff U, Bechtel M and Kramer MD (1996) $\alpha_2$-Antiplasmin and plasminogen activator inhibitors in healing human skin wounds. *Arch Dermatol Res* **288**: 122–128

Schraffordt Koops H, Vaglini M, Suciu S, Kroon BBR, Thompson JF, Göhl J, Eggermont AMM, Di Filippo F, Krementz ET, Ruiter DJ and Lejeune FJ (1998) Prophylactic isolated limb perfusion for localized, high-risk limb melanoma: results of a multicenter randomized phases III trial. *J Clin Oncol* **16**: 2906–2912

Shapiro RL, Duquette JG, Roses DF, Nunes I, Harris MN, Kamino H, Wilson EL and Rifkin DB (1996) Induction of primary cutaneous melanocytic neoplasms in urokinase-type plasminogen activator (uPA)-deficient and wild-type mice: cellular blue nevi invade but do not progress to malignant melanoma in uPA-deficient animals. *Cancer Res* **56**: 3597–3604

Takei A, Tashiro Y, Nakashima Y and Sueishi K (1995) Effects of fibrin on the angiogenesis in vitro of bovine endothelial cells in collagen gel. *In Vitro Cell Dev Biol* **31**: 467–472

Takeuchi Y, Nakao A, Harada A, Nonami T, Fukatsu T and Takagi H (1993) Expression of plasminogen activators and their inhibitors in human pancreatic carcinoma: immunohistochemical study. *Am J Gastroenterol* **88**: 1928–1933

Torzewski M, Sarbia M, Verreet P, Dutkowski P, Heep H, Willers R and Gabbert HE (1997) Prognostic significance of urokinase-type plasminogen activator expression in squamous cell carcinomas of the esophagus. *Clin Cancer Res* **3**: 2263–2268

Umeda T, Eguchi Y, Okino K, Kodama M and Hattori T (1997) Cellular localization of urokinase-type plasminogen activator, its inhibitors, and their mRNAs in breast cancer tissues. *J Pathol* **183**: 388–397

Welling TH, Huber TS, Messina LM and Stanley JC (1996) Tissue plasminogen activator increases canine endothelial cell proliferation rate through a plamin-independent, receptor-mediated mechanism. *J Surg Res* **66**: 36–42

Westphal JR, van't Hullenaar R, Geurts-Moespot A, Sweep FCGJ, Verheijen JH, Bussemakers MMG, Askaa J, Eggermont AAM, Ruiter DJ, de Waal RMW (2000) Angiostatin generation by human tumor cell lines: involvement of plasminogen activators. *Int J Cancer* **86**: 760–767

Yamashita J, Ogawa M, Yamashita S, Nakashima T, Saishoji T, Nomura K, Inada K and Kawano I (1993) Differential biological significance of tissue-type and urokinase-type plasminogen activator in human breast cancer. *Br J Cancer* **68**: 524–529

Yamashita J, Ogawa M and Sakai K (1995) Prognostic significance of three novel biologic factors in a clinical trial of adjuvant therapy for node-negative breast cancer. *Surgery* **117**: 601–608

*© 2000 Cancer Research Campaign*

## References

[PDF_00673] Allgayer H., Babic R., Grützner K. U., Beyer B. C., Tarabichi A., Schildberg F. W., Heiss M. M. (1998). Tumor-associated proteases and inhibitors in gastric cancer: analysis of prognostic impact and individual risk protease patterns.. Clin Exp Metastasis.

[PDF_00677] Andreasen P. A., Kjøller L., Christensen L., Duffy M. J. (1997). The urokinase-type plasminogen activator system in cancer metastasis: a review.. Int J Cancer.

[PDF_00680] Berney C. R., Yang J., Fisher R. J., Russell P. J., Crowe P. J. (1998). Correlates of urokinase-type plasminogen activator in colorectal cancer: positive relationship with nm23 and c-erbB-2 protein expression.. Oncol Res.

[PDF_00683] Bindal A. K., Hammoud M., Shi W. M., Wu S. Z., Sawaya R., Rao J. S. (1994). Prognostic significance of proteolytic enzymes in human brain tumors.. J Neurooncol.

[PDF_00686] Cardinali M., Uchino R., Chung S. I. (1990). Interaction of fibrinogen with murine melanoma cells: covalent association with cell membranes and protection against recognition by lymphokine-activated killer cells.. Cancer Res.

[PDF_00690] Chambers S. K., Ivins C. M., Carcangiu M. L. (1997). Expression of plasminogen activator inhibitor-2 in epithelial ovarian cancer: a favorable prognostic factor related to the actions of CSF-1.. Int J Cancer.

[PDF_00693] Chambers S. K., Ivins C. M., Carcangiu M. L. (1998). Plasminogen activator inhibitor-1 is an independent poor prognostic factor for survival in advanced stage epithelial ovarian cancer patients.. Int J Cancer.

[PDF_00704] De Vries T. J., Mooy C. M., Van Balken M. R., Luyten G. P., Quax P. H., Verspaget H. W., Weidle U. H., Ruiter D. J., Van Muijen G. N. (1995). Components of the plasminogen activation system in uveal melanoma--a clinico-pathological study.. J Pathol.

[PDF_00697] Delbaldo C., Masouye I., Saurat J. H., Vassalli J. D., Sappino A. P. (1994). Plasminogen activation in melanocytic neoplasia.. Cancer Res.

[PDF_00719] Duffy M. J., Maguire T. M., McDermott E. W., O'Higgins N. (1999). Urokinase plasminogen activator: a prognostic marker in multiple types of cancer.. J Surg Oncol.

[PDF_00716] Duffy M. J., O'Grady P., Devaney D., O'Siorain L., Fennelly J. J., Lijnen H. R. (1988). Tissue-type plasminogen activator, a new prognostic marker in breast cancer.. Cancer Res.

[PDF_00722] Duggan C., Kennedy S., Kramer M. D., Barnes C., Elvin P., McDermott E., O'Higgins N., Duffy M. J. (1997). Plasminogen activator inhibitor type 2 in breast cancer.. Br J Cancer.

[PDF_00725] Engelberg H. (1999). Actions of heparin that may affect the malignant process.. Cancer.

[PDF_00733] Ferrier C. M., de Witte H. H., Straatman H., van Tienoven D. H., van Geloof W. L., Rietveld F. J., Sweep C. G., Ruiter D. J., van Muijen G. N. (1999). Comparison of immunohistochemistry with immunoassay (ELISA) for the detection of components of the plasminogen activation system in human tumour tissue.. Br J Cancer.

[PDF_00727] Ferrier C. M., van Geloof W. L., de Witte H. H., Kramer M. D., Ruiter D. J., van Muijen G. N. (1998). Epitopes of components of the plasminogen activation system are re-exposed in formalin-fixed paraffin sections by different retrieval techniques.. J Histochem Cytochem.

[PDF_00731] Ferrier C. M., van Muijen G. N., Ruiter D. J. (1998). Proteases in cutaneous melanoma.. Ann Med.

[PDF_00738] Francis J. L., Biggerstaff J., Amirkhosravi A. (1998). Hemostasis and malignancy.. Semin Thromb Hemost.

[PDF_00740] Ganesh S., Sier C. F., Heerding M. M., van Krieken J. H., Griffioen G., Welvaart K., van de Velde C. J., Verheijen J. H., Lamers C. B., Verspaget H. W. (1996). Prognostic value of the plasminogen activation system in patients with gastric carcinoma.. Cancer.

[PDF_00744] Grebenschikov N., Geurts-Moespot A., De Witte H., Heuvel J., Leake R., Sweep F., Benraad T. (1997). A sensitive and robust assay for urokinase and tissue-type plasminogen activators (uPA and tPA) and their inhibitor type I (PAI-1) in breast tumor cytosols.. Int J Biol Markers.

[PDF_00748] Gunji Y., Gorelik E. (1988). Role of fibrin coagulation in protection of murine tumor cells from destruction by cytotoxic cells.. Cancer Res.

[PDF_00750] Hsu D. W., Efird J. T., Hedley-Whyte E. T. (1995). Prognostic role of urokinase-type plasminogen activator in human gliomas.. Am J Pathol.

[PDF_00752] Hu G., Riordan J. F., Vallee B. L. (1994). Angiogenin promotes invasiveness of cultured endothelial cells by stimulation of cell-associated proteolytic activities.. Proc Natl Acad Sci U S A.

[PDF_00755] Huijzer J. C., Uhlenkott C. E., Meadows G. G. (1995). Differences in expression of metalloproteinases and plasminogen activators in murine melanocytes and B16 melanoma variants: lack of association with in vitro invasion.. Int J Cancer.

[PDF_00759] Ito H., Yonemura Y., Fujita H., Tsuchihara K., Kawamura T., Nojima N., Fujimura T., Nose H., Endo Y., Sasaki T. (1996). Prognostic relevance of urokinase-type plasminogen activator (uPA) and plasminogen activator inhibitors PAI-1 and PAI-2 in gastric cancer.. Virchows Arch.

[PDF_00763] Jänicke F., Schmitt M., Graeff H. (1991). Clinical relevance of the urokinase-type and tissue-type plasminogen activators and of their type 1 inhibitor in breast cancer.. Semin Thromb Hemost.

[PDF_00771] Kim S. J., Shiba E., Kobayashi T., Yayoi E., Furukawa J., Takatsuka Y., Shin E., Koyama H., Inaji H., Takai S. (1998). Prognostic impact of urokinase-type plasminogen activator (PA), PA inhibitor type-1, and tissue-type PA antigen levels in node-negative breast cancer: a prospective study on multicenter basis.. Clin Cancer Res.

[PDF_00768] Kim S. J., Shiba E., Taguchi T., Watanabe T., Tanji Y., Kimoto Y., Izukura M., Takai S. I. (1997). Urokinase type plasminogen activator receptor is a novel prognostic factor in breast cancer.. Anticancer Res.

[PDF_00776] Kim S. J., Shiba E., Tsukamoto F., Izukura M., Taguchi T., Yoneda K., Tanji Y., Kimoto Y., Takai S. I. (1998). The expression of urokinase type plasminogen activator is a novel prognostic factor in dukes B and C colorectal cancer.. Oncol Rep.

[PDF_00780] Kobayashi H., Fujishiro S., Terao T. (1994). Impact of urokinase-type plasminogen activator and its inhibitor type 1 on prognosis in cervical cancer of the uterus.. Cancer Res.

[PDF_00851] Koops H. S., Vaglini M., Suciu S., Kroon B. B., Thompson J. F., Göhl J., Eggermont A. M., Di Filippo F., Krementz E. T., Ruiter D. (1998). Prophylactic isolated limb perfusion for localized, high-risk limb melanoma: results of a multicenter randomized phase III trial. European Organization for Research and Treatment of Cancer Malignant Melanoma Cooperative Group Protocol 18832, the World Health Organization Melanoma Program Trial 15, and the North American Perfusion Group Southwest Oncology Group-8593.. J Clin Oncol.

[PDF_00783] Lansink M., Koolwijk P., van Hinsbergh V., Kooistra T. (1998). Effect of steroid hormones and retinoids on the formation of capillary-like tubular structures of human microvascular endothelial cells in fibrin matrices is related to urokinase expression.. Blood.

[PDF_00787] Markus G. (1984). The role of hemostasis and fibrinolysis in the metastatic spread of cancer.. Semin Thromb Hemost.

[PDF_00789] Meissauer A., Kramer M. D., Schirrmacher V., Brunner G. (1992). Generation of cell surface-bound plasmin by cell-associated urokinase-type or secreted tissue-type plasminogen activator: a key event in melanoma cell invasiveness in vitro.. Exp Cell Res.

[PDF_00793] Mueller B. M. (1996). Different roles for plasminogen activators and metalloproteinases in melanoma metastasis.. Curr Top Microbiol Immunol.

[PDF_00796] Mulcahy H. E., Duffy M. J., Gibbons D., McCarthy P., Parfrey N. A., O'Donoghue D. P., Sheahan K. (1994). Urokinase-type plasminogen activator and outcome in Dukes' B colorectal cancer.. Lancet.

[PDF_00799] Nakanishi K., Kawai T., Torikata C., Aurues T., Ikeda T. (1998). Urokinase-type plasminogen activator, its inhibitor, and its receptor in patients with upper urinary tract carcinoma.. Cancer.

[PDF_00802] O'Reilly M. S., Holmgren L., Chen C., Folkman J. (1996). Angiostatin induces and sustains dormancy of human primary tumors in mice.. Nat Med.

[PDF_00804] Pappot H., Skov B. G., Pyke C., Grøndahl-Hansen J. (1997). Levels of plasminogen activator inhibitor type 1 and urokinase plasminogen activator receptor in non-small cell lung cancer as measured by quantitative ELISA and semiquantitative immunohistochemistry.. Lung Cancer.

[PDF_00808] Quax P. H., van Muijen G. N., Weening-Verhoeff E. J., Lund L. R., Danø K., Ruiter D. J., Verheijen J. H. (1991). Metastatic behavior of human melanoma cell lines in nude mice correlates with urokinase-type plasminogen activator, its type-1 inhibitor, and urokinase-mediated matrix degradation.. J Cell Biol.

[PDF_00812] Reuning U., Magdolen V., Wilhelm O., Fischer K., Lutz V., Graeff H., Schmitt M. (1998). Multifunctional potential of the plasminogen activation system in tumor invasion and metastasis (review).. Int J Oncol.

[PDF_00815] Rickles F. R., Edwards R. L. (1983). Activation of blood coagulation in cancer: Trousseau's syndrome revisited.. Blood.

[PDF_00827] Ruiter D. J., Ferrier C. M., van Muijen G. N., Henzen-Logmans S. C., Kennedy S., Kramer M. D., Nielsen B. S., Schmitt M. (1998). Quality control of immunohistochemical evaluation of tumour-associated plasminogen activators and related components. European BIOMED-1 Concerted Action on Clinical Relevance of Proteases in Tumour Invasion and Metastasis.. Eur J Cancer.

[PDF_00831] Ruiter D. J., van Muijen G. N. (1998). Markers of melanocytic tumour progression.. J Pathol.

[PDF_00833] Ruppert C., Ehrenforth S., Scharrer I., Halberstadt E. (1997). Protease levels in breast, ovary, and other gynecological tumor tissues: prognostic importance in breast cancer.. Cancer Detect Prev.

[PDF_00817] Rønne E., Behrendt N., Ellis V., Ploug M., Danø K., Høyer-Hansen G. (1991). Cell-induced potentiation of the plasminogen activation system is abolished by a monoclonal antibody that recognizes the NH2-terminal domain of the urokinase receptor.. FEBS Lett.

[PDF_00822] Rønne E., Høyer-Hansen G., Brünner N., Pedersen H., Rank F., Osborne C. K., Clark G. M., Danø K., Grøndahl-Hansen J. (1995). Urokinase receptor in breast cancer tissue extracts. Enzyme-linked immunosorbent assay with a combination of mono- and polyclonal antibodies.. Breast Cancer Res Treat.

[PDF_00836] Sakamoto T., Spee C., Scuric Z., Gordon E. M., Hinton D. R., Anderson W. F., Ryan S. J. (1998). Ability of retroviral transduction to modify the angiogenic characteristics of RPE cells.. Graefes Arch Clin Exp Ophthalmol.

[PDF_00843] Sato T., Nishimura G., Yonemura Y., Nojima N., Ninomiya I., Fujimura T., Sugiyama K., Miwa K., Miyazaki I., Nonomura A. (1995). Association of immunohistochemical detection of urokinase-type plasminogen activator with metastasis and prognosis in colorectal cancer.. Oncology.

[PDF_00839] Sato Y., Okamura K., Morimoto A., Hamanaka R., Hamaguchi K., Shimada T., Ono M., Kohno K., Sakata T., Kuwano M. (1993). Indispensable role of tissue-type plasminogen activator in growth factor-dependent tube formation of human microvascular endothelial cells in vitro.. Exp Cell Res.

[PDF_00847] Schaefer B. M., Maier K., Eickhoff U., Bechtel M., Kramer M. D. (1996). alpha 2-Antiplasmin and plasminogen activator inhibitors in healing human skin wounds.. Arch Dermatol Res.

[PDF_00856] Shapiro R. L., Duquette J. G., Roses D. F., Nunes I., Harris M. N., Kamino H., Wilson E. L., Rifkin D. B. (1996). Induction of primary cutaneous melanocytic neoplasms in urokinase-type plasminogen activator (uPA)-deficient and wild-type mice: cellular blue nevi invade but do not progress to malignant melanoma in uPA-deficient animals.. Cancer Res.

[PDF_00861] Takei A., Tashiro Y., Nakashima Y., Sueishi K. (1995). Effects of fibrin on the angiogenesis in vitro of bovine endothelial cells in collagen gel.. In Vitro Cell Dev Biol Anim.

[PDF_00864] Takeuchi Y., Nakao A., Harada A., Nonami T., Fukatsu T., Takagi H. (1993). Expression of plasminogen activators and their inhibitors in human pancreatic carcinoma: immunohistochemical study.. Am J Gastroenterol.

[PDF_00867] Torzewski M., Sarbia M., Verreet P., Dutkowski P., Heep H., Willers R., Gabbert H. E. (1997). Prognostic significance of urokinase-type plasminogen activator expression in squamous cell carcinomas of the esophagus.. Clin Cancer Res.

[PDF_00871] Umeda T., Eguchi Y., Okino K., Kodama M., Hattori T. (1997). Cellular localization of urokinase-type plasminogen activator, its inhibitors, and their mRNAs in breast cancer tissues.. J Pathol.

[PDF_00874] Welling T. H., Huber T. S., Messina L. M., Stanley J. C. (1996). Tissue plasminogen activator increases canine endothelial cell proliferation rate through a plasmin-independent, receptor-mediated mechanism.. J Surg Res.

[PDF_00878] Westphal J. R., Van't Hullenaar R., Geurts-Moespot A., Sweep F. C., Verheijen J. H., Bussemakers M. M., Askaa J., Clemmensen I., Eggermont A. A., Ruiter D. J. (2000). Angiostatin generation by human tumor cell lines: involvement of plasminogen activators.. Int J Cancer.

[PDF_00885] Yamashita J., Ogawa M., Sakai K. (1995). Prognostic significance of three novel biologic factors in a clinical trial of adjuvant therapy for node-negative breast cancer.. Surgery.

[PDF_00881] Yamashita J., Ogawa M., Yamashita S., Nakashima Y., Saishoji T., Nomura K., Inada K., Kawano I. (1993). Differential biological significance of tissue-type and urokinase-type plasminogen activator in human breast cancer.. Br J Cancer.

[PDF_00700] de Vries T. J., Quax P. H., Denijn M., Verrijp K. N., Verheijen J. H., Verspaget H. W., Weidle U. H., Ruiter D. J., van Muijen G. N. (1994). Plasminogen activators, their inhibitors, and urokinase receptor emerge in late stages of melanocytic tumor progression.. Am J Pathol.

[PDF_00708] de Vries T. J., van Muijen G. N., Ruiter D. J. (1996). The plasminogen activation system in melanoma cell lines and in melanocytic lesions.. Melanoma Res.

[PDF_00711] de Witte J. H., Sweep C. G., Klijn J. G., Grebenschikov N., Peters H. A., Look M. P., van Tienoven T. H., Heuvel J. J., Bolt-De Vries J., Benraad T. J. (1999). Prognostic value of tissue-type plasminogen activator (tPA) and its complex with the type-1 inhibitor (PAI-1) in breast cancer.. Br J Cancer.

